# When directional selection reduces geographic variation in traits mediating species interactions

**DOI:** 10.1002/ece3.518

**Published:** 2013-03-05

**Authors:** C W Benkman, T L Parchman

**Affiliations:** 1 Department of Zoology and Physiology, University of WyomingLaramie, Wyoming 82071, USA; 2 Department of Botany, University of WyomingLaramie, Wyoming 82071, USA

**Keywords:** Coevolutionary alternation with escalation, habitat choice, *Loxia curvirostra*, phenotypic selection, *Pinus nigra*, seed predation

## Abstract

Although we often focus on the causes of geographic variation, understanding processes that act to reduce geographic variation is also important. Here, we consider a process whereby adaptive foraging across the landscape and directional selection exerted by a conifer seed predator, the common crossbill (*Loxia curvirostra*), potentially act to homogenize geographic variation in the defensive traits of its prey. We measured seed predation and phenotypic selection exerted by crossbills on black pine (*Pinus nigra*) at two sites in the Pindos Mountains, Greece. Seed predation by crossbills was over an order of magnitude higher at the site where cone scale thickness was significantly thinner, which was also the cone trait that was the target of selection at the high predation site. Additional comparisons of selection differentials demonstrate that crossbills exert selection on black pine that is consistent in form across space and time, and increases in strength with increasing seed predation. If predators distribute themselves in relation to the defensive traits of their prey and the strength of selection predators exert is proportional to the amount of predation, then predators may act to homogenize trait variation among populations of their prey in a process analogous to coevolutionary alternation with escalation.

## Introduction

Many recent studies of coevolution have found geographic variation in the form of selection or in the traits at the phenotypic interface of the interaction (e.g. Benkman 1999; Brodie et al. 2002; Thompson and Cunningham 2002; Toju and Sota 2006; Siepielski and Benkman 2007; Toju 2007; Gómez et al. 2009). These studies, framed in the context of the geographic mosaic theory of coevolution (Thompson 2005), have emphasized geographic variation in the mechanisms giving rise to phenotypic variation. This is understandable as characterizing geographic patterns of variation and elucidating the processes driving this variation are fundamental problems in evolutionary biology (Coyne and Orr 2004; Thompson 2005). However, genetic and phenotypic divergence is not the only outcome of geographically variable processes. Indeed, trait variation can be relatively limited across large areas, implying a role for processes such as gene flow and stabilizing selection that inhibit the evolution of geographic variation. Consequently, understanding when and why divergence has not occurred is also important.

**Figure 1:**
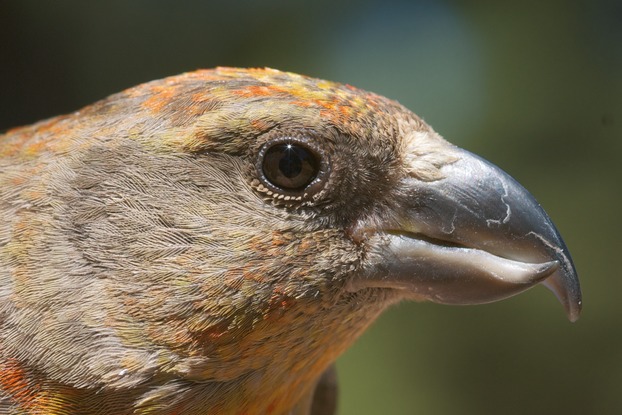
Head of a male common crossbill (*Loxia curvirostra*). The crossed mandibles are essential for biting and forming gaps between closed cone scales so that seeds can be extracted from the base of the scales.

Here, we focus on the predator-prey interactions between crossbills and conifers to address a mechanism that could act to homogenize variation among populations. Crossbills (*Loxia*) are specialized for extracting seeds secured within conifer (Pinaceae) cones (Fig. 1; Newton 1972; Benkman and Lindholm 1991), where the woody cone scales act to defend seeds from seed predators (Smith 1970) such as crossbills (Benkman et al. 2010). Because crossbills forage for seeds in a stereotypic manner (Newton 1972; Benkman 1987b) and preferentially forage on trees whose seeds are most accessible (Benkman 1987a), they exert selection in a consistent manner on certain cone traits (e.g. scale thickness; Benkman et al. 2010, 2013). Increases in these cone traits in turn result in selection favoring larger bills in the crossbills (Benkman 2003; Benkman et al. 2003).

Previous research on crossbill – conifer interactions has examined the processes contributing to geographic variation in coevolutionary interactions and outcomes (Benkman 1999; Benkman et al. 2001, 2003, 2010, 2013; Parchman and Benkman 2002, 2008; Mezquida and Benkman 2005, 2010; Siepielski and Benkman 2005; Parchman et al. 2007; Benkman and Parchman 2009). Although these studies have found striking and repeated patterns of divergence depending on factors influencing the abundance of crossbills and their extent of seed predation (e.g. presence and absence of tree squirrels, conifer forest area, and time), the cone traits of some conifers can be fairly similar across extensive areas. For example, cone structure is quite uniform across much of the vast distribution of Rocky Mountain lodgepole pine (*Pinus contorta* Douglas ex Loudon var. *latifolia* Engelm.) from the Yukon to Colorado (Wheeler and Guries 1982). Although there is often considerable variation within populations, the cone size distributions exhibit relatively little variation among populations except in isolated mountain ranges where pine squirrels are absent (Benkman 1999).

Due to large effective populations sizes and high levels of outcrossing, conifer populations are typically characterized by a lack of neutral genetic differentiation (Neale and Savolainen 2004; Savolainen et al. 2007). However, conifers are also noted for considerable fine-scale adaptive variation in spite of gene flow (Petit et al. 2004; Savolainen et al. 2007). Cone traits in particular have repeatedly been found to be under phenotypic selection and to respond rather rapidly to such selection (e.g. Benkman et al. 2003, 2010; Siepielski and Benkman 2007). Thus, it is doubtful that gene flow alone is an explanation for the relative uniformity of cone traits across large regions.

A coevolutionary process that could act to reduce variation in traits subject to selection among locations is coevolutionary alternation with escalation (Thompson 2005). This process was originally described for a single predator species coevolving with multiple prey species whereby the predator actively searches for prey and preferentially attacks the more profitable prey species favoring the escalation of its defenses (Thompson 2005). As the defenses of the more vulnerable and profitable species increase, the predator includes additional species in its diet until eventually all prey species are equally defended and profitable to the predator. This mechanism provides a plausible explanation for the elevated defenses of multiple species of snails in response to the enhanced counter-defenses of crabs in Lake Tanganyika (West et al. 1991; West and Cohen 1996; Thompson 2005). A similar but undescribed process could occur among populations within a single prey species, if the predator is mobile and distributes itself in relation to the vulnerability of its prey. Whether crossbills distribute themselves among areas in relation to variation in seed defenses (accessibility of seeds) within a single species of conifer is unknown but likely given that crossbills (1) are highly mobile (may fly multiple km within a day and 1000s of km yearly in search of large seed crops that vary in location from year to year) and switch from feeding on one conifer to another (both within and between habitats) as their relative profitabilities shift (Benkman 1987a), and (2) forage preferentially on trees within a habitat whose seeds are most accessible (Benkman et al. 2010).

**Figure 2:**
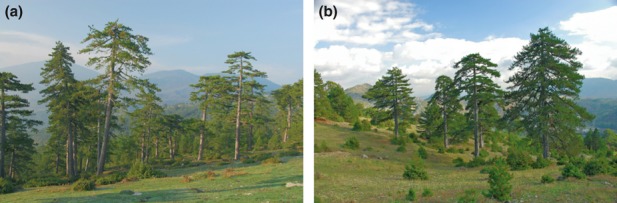
Photographs of the black pine (*Pinus nigra*) forests at the two sites, Samarina (a) and Aetia (b), to illustrate generally similar forest structure.

To address whether the extent of seed predation by common crossbills (*L. curvirostra* L.) varies in relation to variation in the level of seed defenses among sites we present data from two areas of European black pine (*Pinus nigra* J.F. Arnold) in the Pindos Mountains, Greece. An earlier study on crossbills and black pine focused on how the presence and absence of tree squirrels and variation in forest area contribute to geographic variation in cone traits (Benkman and Parchman 2009). We assume that the extent of seed predation is a metric of crossbill abundance and habitat use because crossbills rely almost exclusively on seeds in conifer cones (see Benkman et al. 2013; for evidence of positive correlations between seed predator abundance and pine seed predation). Although the two sites were only 5 km apart and appeared similar in forest structure (Fig. 2), seed predation by crossbills was high at one site but surprisingly low at the other site especially given that 5 km is well within a crossbill's daily cruising range (C. W. Benkman, pers. obs.). We found that the traits under direct selection (target of selection) and deter crossbills were elevated in the underused site, which implies that the difference in site use and seed predation by crossbills was the result of differences between sites in cone traits that deter crossbills. These results provide a mechanism for the escalation and homogenization of defenses across a landscape, and illustrate how the conditions influencing one process, a coevolutionary arms race, account for trait uniformity in some situations and trait divergence between populations in other situations.

## Materials and Methods

To quantify total seed predation, cone traits, and phenotypic selection, we estimated the proportion of the seed crop consumed by crossbills and quantified cone traits for 78 trees at the high predation site near Samarina (1490 m elev., N 40º05.763′, E 21º07.568) and for 97 trees at the low predation site 5-km to the southeast near Aetia (1161 m elevation, N 40º04.483′, E 21º10.622′) between 7 and 11 November 2008. Both sites were on southwest-facing slopes with open forests dominated by black pine (Fig. 2). Black pine seeds and cones grow during summer with seeds maturing by September. Cones remain closed until spring when they open and shed their seeds (Skordilis and Thanos 1997). Crossbills begin foraging on the cones in July and spread apart and often shred the cone scales to reach the underlying seeds (Fig. 1). After foraging, they drop the cones to the ground, making predation readily quantifiable. Although our measures of seed predation represent only a fraction of the total seed predation the trees would experience over the year, they should be representative of both the differences in seed predation between the sites and the general form of phenotypic selection exerted by crossbills on cone structure, because cone structure does not change over most of the time during which seed predation by crossbills occurs including much of the period prior to our measurement of seed predation. An evolutionary response to selection is expected, as cone traits of black pine are highly heritable (e.g. family heritabilities of 0.92 and 0.98 for cone mass and seed mass, respectively; Kaya and Temerit 1993).

Trees were selected haphazardly with the constraint that we could collect at least three closed cones using a 9-m long pole with a clipper at the end. For each tree, we measured the diameter at breast height (DBH) with a measuring tape and counted the number of cones on half of the tree using 10 × 42 binoculars (distinguishing cones remaining on the tree that had been foraged on by crossbills); this value was doubled to provide an estimate of the total number of cones on the tree. We gathered and counted all shredded cones that had been removed by crossbills and had fallen to the ground. Red squirrels (*Sciurus vulgaris* L.) also foraged on cones, and we recorded the number of cone cores left by foraging squirrels. Because squirrels foraged on relatively few cones (e.g. 0.3 cones/tree or 0.3% of the cones in Samarina and 1.4 cones/tree or 0.1% of the cones in Aetia), we do not include analyses of the cone cores other than to include them in the total number of cones produced per tree. The number of shredded cones divided by the total number of cones was the proportion of seeds eaten by crossbills, as crossbills removed and consumed all or nearly all seeds from the shredded cones (C. W. Benkman & T. L. Parchman, pers. obs.).

We used the percentage of seeds (cones) not eaten as a surrogate for tree fitness (e.g. Benkman et al. 2003; Siepielski and Benkman 2007; Benkman and Parchman 2009). Although the total number of seeds produced in a lifetime is a preferred metric, we believe the percentage of seeds not eaten is suitable for long-lived trees for which we are measuring only a single seed crop and where the number of seeds produced varies with tree size and age (e.g. at Samarina the number of cones produced was correlated with tree DBH: *r* = 0.32, *n* = 78 trees, *P* = 0.004). A tree that produces cones with traits that deter crossbills so that few seeds are eaten will always be assigned a high fitness if we used percent of seeds not eaten (Siepielski and Benkman 2007). In contrast, assigned fitness would largely depend on the tree's size/age relative to other trees when the study was conducted, if we used total number of seeds not eaten. Using the proportion of seeds, however, could be problematic if, for example, seed predators avoided large cones and there was trade-off between cone size and the number of seeds per cone. Such a trade-off was not evident for black pine. Instead, we found a positive correlation between individual cone mass and the number of full (filled with female gametophyte) seeds at each site (Samarina: *r* = 0.39, *P* = 0.0004; Aetia: *r* = 0.25, *P* = 0.01), as we have typically found in other pines (Benkman et al. 2003; Mezquida and Benkman 2005; Parchman and Benkman 2008).

We measured cone length and width for 3–7 closed cones collected from each tree (mean = 4.0 and 3.3 for Samarina and Aetia, respectively) before opening cones in a drying oven. The following measurements were taken for each opened cone: cone mass without seeds, thicknesses of six scales selected approximately equidistant around the proximal end of the cone, the length of six scales selected approximately equidistant around the distal end of the cone, the number of full and empty seeds, and the individual masses of five full seeds (see Benkman et al. 2003 for additional information on cone measurements). All length measurements were made to the nearest 0.01 mm with digital calipers. All mass measurements were made to the nearest 0.01 mg with a digital scale after the cones had been oven-dried at 60°C for >2 days. The mean values of trees for each trait were the unit of analysis.

### Analyses of cone traits, seed predation, and selection

We tested for differences in the various cone and tree traits (ln-transformed) between the two sites using *t*-tests or Kruskal–Wallis tests depending on whether the assumptions of normality and equal variances were met for using *t*-tests. We used multiple linear regressions and their regression coefficients to estimate selection gradients (*β*) and identify the traits under direct selection (i.e. the targets of selection; Lande and Arnold 1983). We also used these regressions to evaluate which features contribute to the difference in overall seed predation by crossbills between sites. To avoid problems with multicollinearity in the multiple regression models, we examined correlation coefficients (*r*) between traits and variance inflation factor (VIF) scores. We only included traits with |*r*| < 0.38 and VIF scores <2; the same set of traits was included in each multiple regression. Relative tree fitness was estimated as 100 minus percent seed predation divided by the overall mean percent seed predation in each population, and cone traits were standardized to units of standard deviations. We excluded one tree from Aetia from the analyses of selection because it was an extreme outlier (*P* < 0.001) in terms of relative fitness. We used the regression bootstrap to estimate the standard errors and 95% confidence intervals for the selection gradients because the residuals in the regressions were strongly non-normally distributed. These estimates were based on 1000 bootstrap replicates, and these analyses were run in JMP® Pro 10.0.0 (SAS Institute Inc., Cary, NC).

We also evaluated whether the selection (both direct and indirect selection) we measured is likely representative of selection experienced in the population over a longer interval and similar among multiple sites, which are necessary for trait homogeneity to arise from coevolutionary alternation with escalation. This was done by performing separate least squares regressions comparing the standardized linear selection differentials (*s;* Lande and Arnold 1983) to those measured in an identical manner in other sites. The other sites included 96 black pine trees in the Troodos Mountains, Cyprus on 13–16 November 2008 and those from a published study conducted on crossbills and black pine in the laboratory and in the Troodos Mountains on 10–16 January 2006 (Benkman and Parchman 2009). We also estimated standardized quadratic selection gradients (γ; Lande and Arnold 1983) and present those that were significant (*P* < 0.05). However, we did not include the quadratic selection gradients in the comparative analyses; for three cases where the quadratic term was significant, analyses using cubic splines showed that the form of selection was directional. In addition, previous analyses of selection exerted by crossbills indicate that selection when present is consistently directional (Benkman et al. 2010). We included two composite variables: the ratio of seed mass to cone mass (a measure of the amount of energy devoted to reproduction relative to seed defense) and the ratio of cone width to cone length (a measure of cone shape). Seed mass is the product of mean individual seed mass multiplied by the mean number of full seeds per cone. We estimated relative fitness, standardized cone traits, used the same regression bootstrap technique, and estimated the standard errors and 95% confidence intervals for the selection differentials, as done for the selection gradients using JMP® Pro 10.0.0.

A significant positive relationship between the linear selection differentials among the different datasets would indicate similarity in selection. We used nonparametric bootstraps (*n* = 1000) to calculate confidence intervals for the regression coefficients to account for uncertainty in the estimates of the selection differentials and because the residual distributions of these regressions were unknown. For each bootstrap replicate, we resampled the original data from each dataset, calculated the selection differentials for each trait in the resampled datasets (as above), and then performed a linear regression on the bootstrap selection differentials for the two sites. We also calculated *P*-values on the hypothesis that the coefficient *b*_*i*_ = 0 using the method described in Davison and Hinkley (1997). Tests of significance were based on 1000 bootstrap replicates, and these analyses were run in R (R development team).

To further explore the variation in the strength of selection among sites, we used regression to test whether selection differentials increased with the percent of seeds eaten. Because the opportunity for selection, or variance in relative fitness, sets the upper limit for the intensity of selection (Crow 1958; Arnold and Wade 1984) and increases with the strength of an antagonistic interaction such as seed predation (Benkman, in review), selection differentials should increase with increasing seed predation if crossbills exhibit similar cone preferences at each site. Moreover, the maximum selection differential is equal to the square root of the opportunity for selection (Arnold and Wade 1984). Thus, we also used regression to determine whether the selection differentials were related to the square root of the opportunity for selection. The opportunity for selection is calculated as Σ (relative fitness – mean relative fitness)^2^/*n*, where *n* is the sample size.

Finally, our measures of selection are based on correlations rather than experiments (with the exception of the earlier laboratory study), which can lead to biased estimates of selection if, for example, local environmental conditions influence both fitness (the probability that seeds are not eaten) and the phenotype of the cones and seeds (Rausher 1992). Such biases, however, are unlikely to be substantial for crossbills based on the similarity between selection differentials measured in this study and those measured in an earlier study conducted in the laboratory (Benkman and Parchman 2009; see Results). In addition, many previous studies documenting the form of selection crossbills exert on conifers have implicated the same traits and have led to a mechanistic understanding of the factors determining seed predation by crossbills (see Benkman et al. 2010 for a review), which strengthens inferences of the causal link between phenotype and fitness (MacColl 2011).

## Results

The proportion of seeds eaten by crossbills was over 10 times higher at Samarina than Aetia, and trees at Samarina were smaller (DBH) and had smaller cones (cone mass) with thinner scales and smaller seeds (Table 1). The differences in the occurrence of crossbills at the two sites appeared even greater (C. W. Benkman & T. L. Parchman, pers. obs.), implying that when crossbills first arrived they sampled both sites, and then all or nearly all crossbills concentrated their foraging activities at Samarina. A multiple regression model for Samarina that included DBH, the number of cones per tree, and several cone traits revealed that the targets of selection were scale thickness, the number of empty seeds, and seed size with crossbills preferentially foraging on trees having cones with thinner scales, fewer empty and smaller seeds (Table 2). This suggests that the higher level of seed predation at Samarina than Aetia was not the result of trees being smaller at Samarina or having fewer empty seeds, but because the trees at Samarina had on average thinner cone scales and smaller seeds (Table 1). This result is further supported by the multiple regression model for Aetia that indicated that crossbills preferentially foraged on larger trees (Table 2). Crossbills would have been expected to forage on smaller trees if the preference for Samarina over Aetia was based on tree size. No individual cone trait was correlated with DBH at both sites (*P* > 0.4 in at least one site for every trait; only one trait, seed mass, was significantly correlated with DBH at either site [*P* = 0.014 at Samarina, but *P* = 0.82 at Aetia]), therefore the differences in cone traits between sites were unlikely to be the result of ontogenetic changes in cone traits.

**Table 1:** Mean (± SE) values for various characteristics of black pine sampled haphazardly at two sites, Samarina and Aetia (*n* = 78 and 97 trees, respectively)

Trait	Samarina	Aetia	*t/χ*^2^	*P*
	Mean ± SE	Mean ± SE		
DBH (cm)	75.90 ± 1.49	96.98 ± 1.57	−9.79	<**0.0001**
Number of cones	312.5 ± 24.2	313.8 ± 22.4	1.70	0.09
Cone length (mm)	61.84 ± 0.79	61.23 ± 0.67	0.57	0.57
Cone width (mm)	31.47 ± 0.37	31.40 ± 0.28	0.06	0.95
Cone width/length	0.511 ± 0.004	0.516 ± 0.005	−0.72	0.47
Cone mass (gm)	14.73 ± 0.48	15.81 ± 0.37	−2.28	**0.02**
Scale thickness (mm)	3.51 ± 0.04	3.64 ± 0.04	−2.27	**0.02**
Scale length (mm)	17.29 ± 0.27	17.71 ± 0.22	−1.27	0.21
Number of full seeds	31.47 ± 1.47	30.82 ± 1.04	0.002	0.96
Number of empty seeds	16.18 ± 0.98	14.87 ± 0.70	0.75	0.46
Individual seed mass (mg)	21.7 ± 4.0	23.5 ± 3.5	−3.02	**0.02**
Seed mass/cone mass	0.0255 ± 0.0088	0.0263 ± 0.0083	0.34	0.56
Percent of seeds predated	11.2 (median = 3.2)	0.9 (median = 0)	67.03	**<0.0001**

*T*-tests were used to test whether the means differed (on ln-transformed data) and the *t* statistics and *P*-values are given, except for the number of full seeds, seed mass/cone mass, and percent seed predation where Kruskal–Wallis tests were used and *χ*^2^ statistic is presented. Significant *P*-values (<0.05) are in bold.

**Table 2:** Selection gradients (*β*) from multiple linear regressions for selection exerted on black pine by crossbills at two sites, Samarina and Aetia (*n* = 78 and 96 trees, respectively)

	Samarina		Aetia	
		
Trait	*β* ± SE	95% CI	*β* ± SE	95% CI
Number of cones	−0.0089 ± 0.0299	−0.0760, 0.0437	−**0.0032** ± 0.0019	−0.0076, −0.0005
DBH	−0.0162 ± 0.0355	−0.0856, 0.0533	**0.0039** ± 0.0017	0.0014, 0.0079
Cone width/length	−0.0098 ± 0.0216	−0.0537, 0.0312	−0.0016 ± 0.0008	−0.0033, 0.0001
Scale thickness	**0.0599** ± 0.0186	0.0247, 0.0985	0.0003 ± 0.0011	−0.0019, 0.0025
Number of full seeds	−0.0149 ± 0.0177	−0.0486, 0.0205	0.0018 ± 0.0010	−0.0001, 0.0039
Number of empty seeds	**0.0339** ± 0.0166	0.0026, 0.0705	**0.0023** ± 0.0011	0.0006, 0.0048
Individual seed mass	**0.0599** ± 0.0233	0.0146, 0.1075	0.0020 ± 0.0013	−0.0002, 0.0049

The overall models for Samarina and Aetia were significant (*P* < 0.0001 and *P* = 0.001, respectively). Selection gradients that do not overlap with the 95% confidence intervals (*P* < 0.05) are in bold. VIF < 2 for all traits in both models, |*r*| < 0.38 for all trait correlations.

**Figure 3:**
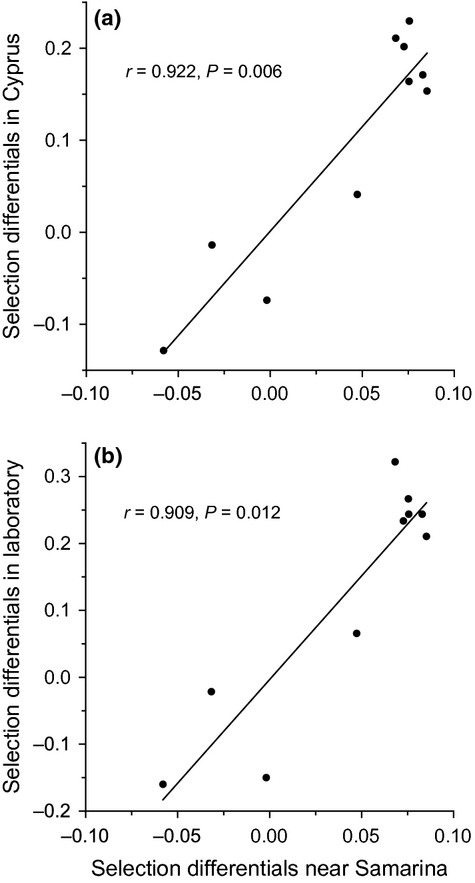
The selection differentials are positively related between those in the Samarina and those in Cyprus in 2008 (A) and in aviary experiments (B). The traits included were all those in Table 3. The lines represent least squares regressions.

The overall form of selection in Samarina, as measured by the selection differentials, was similar to that measured in the laboratory (*r* = 0.909, *P* = 0.012; Fig. 3B), and in Cyprus in both 2006 (*r* = 0.856, *P* = 0.004) and 2008 (*r* = 0.922, *P* = 0.006; Fig. 3A). The relationship was also strong between the 2 years in Cyprus (*r* = 0.884, *P* < 0.001). These results indicate that the selection we measured in Samarina during 1 year is likely representative of the selection exerted by crossbills on black pine over multiple years at a site and across its range.

**Figure 4:**
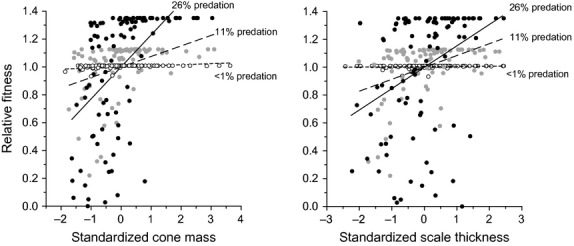
The selection differentials for cone mass and scale thickness increased with increasing levels of seed predation among sites. Less than one percent seed predation represents data from near Aetia, Greece (open circles, short-dashed lines), 11 percent represents data from near Samarina, Greece (gray circles, long-dashed lines), and 26 percent represents data from the Troodos Mountains, Cyprus (black circles, solid lines). The lines represent least squares regressions.

Although the general form of selection was similar among locations and between the laboratory and the field (Fig. 3), the absolute values of the selection differentials varied among field sites and increased with increases in the percent of the seeds eaten by crossbills (Fig. 4; including data from Samarina, Aetia, and the 2 years in Cyprus: S*election differential*_cone mass_ = −0.0021 + 0.0088[*% predation*], *r*^2^ = 0.974, df = 2, *P* = 0.013; S*election differential*_scale thickness_ = −0.0084 + 0.0062[*% predation*], *r*^2^ = 0.885, df = 2, *P* = 0.059; S*election differential*_seed mass_ = 0.0033 + 0.0066[*% predation*], *r*^2^ = 0.996, df = 2, *P* = 0.002). This increase in the selection differentials presumably arises because the variance in relative fitness among trees, and therefore the opportunity for selection, increased as the percent of the seeds eaten increased among Samarina, Aetia, and the 2 years in Cyprus (√*Opportunity for selection* = 0.0164 + 0.0162[*% predation*], *r*^2^ = 0.996, df = 2, *P* = 0.002). This can account for the on average 30 × larger selection differentials at Samarina than at Aetia (Table 3; based on the non-overlapping 95% CIs, all significant selection differentials at Samarina were significantly larger than at Aetia).

**Table 3:** Selection differentials (*s*) for selection exerted by crossbills on various cone traits of black pine at two sites, Samarina and Aetia (*n* = 78 and 96 trees, respectively). Only significant second-order models are shown (quadratic terms were doubled [Lande and Arnold 1983; Stinchcombe et al. 2008])

	Samarina		Aetia	
		
Trait	*s* ± SE	95% CI	*s* ± SE	95% CI
First-order models				
Cone length	**0.0682** ± 0.0186	0.0353, 0.1092	**0.0048** ± 0.0018	0.0020, 0.0095
Cone width	**0.0727** ± 0.0215	0.0379, 0.1214	0.0012 ± 0.0009	−0.0004, 0.0031
Cone width/length	−0.0019 ± 0.0213	−0.0411, 0.0440	−**0.0030** ± 0.0012	−0.0062, −0.0012
Cone mass	**0.0756** ± 0.0201	0.0404, 0.1213	**0.0034** ± 0.0012	0.0017, 0.0064
Scale thickness	**0.0852** ± 0.0248	0.0445, 0.1364	**0.0016** ± 0.0008	0.0003, 0.0037
Scale length	**0.0754** ± 0.0225	0.0350, 0.1211	**0.0028** ± 0.0013	0.0007, 0.0063
Number of full seeds	−0.0317 ± 0.0226	−0.0825, 0.0079	−0.0002 ± 0.0010	−0.0027, 0.0014
Number of empty seeds	**0.0473** ± 0.0195	0.0158, 0.0919	**0.0018** ± 0.0009	0.0002, 0.0038
Individual seed mass	**0.0829** ± 0.0231	0.0438, 0.1315	**0.0023** ± 0.0013	0.0005, 0.0054
Seed mass/cone mass	−**0.0580** ± 0.0283	−0.1219, −0.0111	−0.0018 ± 0.0014	−0.0051, 0.0004
Second-order models				
Cone mass			**0.0056** ± 0.0018	0.0026, 0.0097
[Cone mass]^2^			−**0.0036** ± 0.0015	−0.0068, −0.0010
Scale thickness	**0.0952** ± 0.0289	0.0467, 0.1595		
[Scale thickness]^2^	−**0.0806** ± 0.0384	−0.1622, −0.0157		
Individual seed mass	**0.0907** ± 0.0251	0.0440, 0.1418		
[Individual seed mass]^2^	−**0.0662** ± 0.0260	−0.1214, −0.0178		

Selection differentials that do not overlap zero with the 95% confidence intervals (two-tailed tests, *P* < 0.05) are in bold.

## Discussion

The type and outcome of species interaction documented here, of seed predators preferentially using habitats where seeds are less defended and selection differentials increasing with increasing predation, leads to variation in selection among sites whose strength is inversely proportional to the level of defense. Over time, such selection will elevate defenses but also homogenize them across the landscape. Evolutionary biologists have emphasized gene flow and migration in preventing divergence (Coyne and Orr 2004; Thompson 2005). However, when the enemy̲–victim interaction is one of coevolutionary escalation and the predators (or parasites) are mobile and distribute themselves adaptively in relation to the phenotypes of their victims, then one potential outcome is selection that homogenizes trait values among populations. Below we discuss when predators are likely to distribute themselves (deplete prey) in relation to the defenses (availability) of their prey, why selection differentials increase with increasing predation, and some implications of the above process that could be termed coevolutionary alternation with escalation (Thompson 2005).

Animals often forage adaptively and distribute themselves so that more individuals occur in high quality patches or habitats than in those of lower quality (e.g. ideal free distribution: Tregenza 1995; Hamilton 2010). Many factors can potentially affect the quality of a habitat, but patches where prey are most vulnerable and feeding rates are highest are likely of general importance (Hamilton 2010). In some cases, such as for crossbills, whose feeding rates depend directly on cone morphology, seed accessibility and overall seed intake rates generally dominate habitat choice (Benkman 1987a) and habitat use is likely to be concordant with the phenotypes of victims. This is perhaps more likely for predators that specialize on prey that rely on defenses that make them difficult to process rather than capture. In the case where victim defenses prevent capture, habitat features are likely to affect prey vulnerability and habitat choice for predators. Although we would still expect among patch or habitat variation in the strength of selection exerted by predators, the variation in selection would not necessarily correspond to the distribution of victim phenotypes. Indeed, the diversity of factors affecting habitat choice and local population abundance undoubtedly contributes to the variation in selection often detected among locations (e.g. Thompson and Cunningham 2002; Gómez et al. 2009).

Importantly, because crossbills displayed similar cone preferences in all situations, selection differentials increased progressively with increases in seed predation and the opportunity for selection. Because the maximum selection differential is equal to the square root of the opportunity for selection (Arnold and Wade 1984), the strong relationship between the square root of the opportunity for selection and the selection differentials further demonstrates that the form of selection was similar among sites. Similar cone preferences and thus similar selection exerted by crossbills has been found within other conifers too (e.g. *Pinus ponderosa* [Parchman and Benkman 2008; *Pinus contorta latifolia* [Benkman et al. 2013]; *P. uncinata* [Mezquida & Benkman, in prep.]), which is consistent with crossbill tree use being overwhelmingly related to cone traits rather than some other environmental feature. Nevertheless, populations often experience selection from a variety of abiotic and biotic factors that tend to vary in their relative importance across a landscape that potentially cause geographically divergent selection among populations (Thompson 2005). However, mobility in predators or parasites that distribute themselves adaptively relative to victim defenses could be an important mechanism whereby variation in the strength of directional selection among locations acts to geographically homogenize traits in their victims rather than cause divergence. If the vulnerability of victims is related mostly to their phenotypic traits rather than habitat features, one might predict that traits under selection by enemies should be more similar across populations than traits experiencing selection by other environmental factors.

The above mechanism found for crossbills foraging on black pine is analogous to coevolutionary alternation with escalation, whereby a predator or parasite prefers the least defended victim and adds additional victim species to its diet as relatively poorly defended victim species evolve enhanced defenses (Thompson 2005). Although coevolutionary alternation with escalation was defined as enemies shifting from one victim species to another (Thompson 2005), the same underlying process could occur with shifts between different populations of victims. In the model of coevolutionary alternation (without escalation; Nuismer and Thompson 2006), the predator or parasite specializes on one victim species at a time so that defenses decline in past victim species. This would presumably arise because trade-offs favor specialization on only one or a few victim species at a time. However, in many cases where the variation between different species of victims is less distinct, then predators and parasites are more likely to use multiple species. For the same reason, coevolutionary alternation with escalation is likely to be relatively more common when predators are switching among populations of a single species than of multiple species. In this situation, victim populations are unlikely to lose their defenses and all populations will continue to evolve greater defenses. Although the rate of evolution will vary among populations with the most vulnerable presumably experiencing the strongest selection.

## Conclusions

In sum, because crossbills forage adaptively and often are highly mobile and readily move between forest patches, crossbills may tend to exert selection that homogenizes cone trait variation within a conifer across the landscape. Countering this tendency, however, are multiple factors that limit crossbill abundance in a given area. One such factor is pre-emptive competitors such as tree squirrels (*Tamiasciurus* and *Sciurus*) that reduce the abundance of crossbills and thus reduce the crossbill's impact on cone evolution (Benkman et al. 2010). Another factor is variation in the area of isolated forest patches. Crossbill abundance declines as forest area declines, and therefore the strength of selection exerted by crossbills decreases and cone defenses directed toward crossbills also decline (Siepielski and Benkman 2005; Benkman and Parchman 2009; Mezquida and Benkman 2010). Thus, the resulting geographic mosaic of coevolution between crossbills and conifers arises from the tension between the adaptive foraging behavior of crossbills that tends to homogenize geographic cone trait variation and the various factors creating spatial and consistent heterogeneity in the abundances of crossbills and the selection they exert.
